# A cocoa (*Theobroma cacao* L.) extract impairs the growth, virulence properties, and inflammatory potential of *Fusobacterium nucleatum* and improves oral epithelial barrier function

**DOI:** 10.1371/journal.pone.0252029

**Published:** 2021-05-24

**Authors:** Amel Ben Lagha, Patricia Maquera Huacho, Daniel Grenier

**Affiliations:** Oral Ecology Research Group, Faculty of Dentistry, Université Laval, Quebec City, QC, Canada; University of the Pacific, UNITED STATES

## Abstract

*Fusobacterium nucleatum* is associated with many conditions and diseases, including periodontal diseases that affect tooth-supporting tissues. The aim of the present study was to investigate the effects of a cocoa extract (*Theobroma cacao* L.) on *F*. *nucleatum* with respect to growth, biofilm formation, adherence, and hydrogen sulfide (H_2_S) production. The anti-inflammatory properties and the effect on epithelial barrier function of the cocoa extract were also assessed. The cocoa extract, whose major phenolic compound is epicatechin, dose-dependently inhibited the growth, biofilm formation, adherence properties (basement membrane matrix, oral epithelial cells), and H_2_S production of *F*. *nucleatum*. It also decreased IL-6 and IL-8 production by *F*. *nucleatum*-stimulated oral epithelial cells and inhibited *F*. *nucleatum*-induced NF-κB activation in monocytes. Lastly, the cocoa extract enhanced the barrier function of an oral epithelial model by increasing the transepithelial electrical resistance. We provide evidence that the beneficial properties of an epicatechin-rich cocoa extract may be useful for preventing and/or treating periodontal diseases.

## Introduction

Periodontitis is a multifactorial chronic inflammatory disease that is initiated and maintained by a dysbiotic biofilm and that is characterized by the progressive destruction of the periodontal ligament and alveolar bone [1]. A limited number of Gram-negative anaerobic bacteria with the ability to express virulence factors have been associated with the various stages of periodontitis [2, 3]. Of these periodontal pathogens, *Fusobacterium nucleatum* is believed to play a central role in periodontal biofilm maturation through its ability to act as a bridge between the early (streptococci, actinomyces) and late (*Porphyromonas gingivalis*, *Treponema denticola*, *Tannerella forsythia*) colonizers of oral biofilms [4]. *F*. *nucleatum* has also been associated with extra-oral infections, including appendicitis, osteomyelitis, atherosclerosis, pericarditis, and brain abscesses [5], and may play a role in preterm/low birth weight and colorectal cancer development [5, 6].

*F*. *nucleatum* is known to increase in numbers in diseased periodontal sites [7]. It expresses a number of adhesins that are involved in adherence to salivary proteins, bacteria, and host cells [8–10]. Additional virulence properties associated with *F*. *nucleatum* include hemolytic activity [11] and hydrogen sulfide (H_2_S) production [12]. *F*. *nucleatum* can induce an inflammatory response in different cell types, including oral epithelial cells and macrophages [13, 14]. More specifically, oral epithelial cells challenged with *F*. *nucleatum* respond by producing high levels of cytokines, including interleukin (IL)‐6 and IL‐8 [15, 16].

The gingival epithelium protects the connective and bone tissues from bacterial invasion and thus plays an important role in the innate immune response [17, 18]. In the course of periodontal disease, inflammation caused by bacteria leads to the disruption of epithelial tight junctions, which facilitates bacterial invasion [19]. Translocation of bacteria into the underlying connective tissue promotes a destructive inflammatory response, leading to bone loss [20].

Various therapeutic approaches for treating periodontal disease include surgical intervention, mechanical therapy (ultrasonic debridement, scaling and root planning) and use of pharmacological agents, with the objective to clean the periodontal pockets, polish the root surfaces and kill or remove bacteria. The pharmacological agents, by exerting an antibacterial effect or modulating the host response, are known to enhance treatment outcomes [21]. In this context, plant polyphenols have attracted the interest of several research groups. Cocoa (*Theobroma cacao* L.) is a plant with a high phenolic content and over 300 different constituents [22]. The beneficial effects of cocoa on human health have been mainly attributed to its phenolic compounds, including monomeric flavanols (catechin, epicatechin), dimer procyanidins B2 and B1, and polymeric flavanols [23]. These cocoa phenolic compounds have been described as bioactive natural molecules because of their antioxidant, anti-inflammatory, anticarcinogenic, and cardio-protective properties [22, 24]. While evidence has been brought that cocoa polyphenols may have benefits for cardiovascular diseases, endocrine disorders, and cancers, their impact with respect to periodontal disease has been poorly investigated [22–24].

Given that *F*. *nucleatum* is thought to play a key role in periodontal disease development by modulating the formation of a pathogenic subgingival biofilm that induces chronic inflammation, the aims of the present study were to investigate a cocoa extract for its ability to inhibit the growth, biofilm formation, adherence properties, and H_2_S production of *F*. *nucleatum*, and to attenuate the *F*. *nucleatum*-induced inflammatory response in oral epithelial cell and monocyte models. The ability of the cocoa extract to enhance epithelial barrier function was also assessed.

## Materials and methods

### Cocoa extract and phenolic characterization

A cocoa extract prepared by ethanol/water extraction of cocoa beans (*Theobroma cacao* L.) was kindly provided by Naturex (Avignon, France). A stock solution (20 mg/mL) was prepared in 10% dimethylsulfoxide (DMSO), sterilized by filtration (0.22 μm pore size), and kept at 4°C protected from light. Anthocyanins and procyanidins in the extract were characterized by high-performance liquid chromatography (HPLC) as described previously [25]. Delphinidin 3-glucoside was used as a standard for anthocyanins. Procyanidins, which were eluted on the basis of their degree of polymerization, were quantified using epicatechin monomer as a standard. Phenolic acids and flavonoids were characterized as previously described [25] using an Acquity^®^ ultra-performance liquid chromatography-tandem mass spectrometer (UHPLC–MS/MS) coupled to a triple quadrupole (TQD) mass spectrometer equipped with a Z-spray electrospray interface (Waters Ltd., Mississauga, ON, Canada). External phenolic and flavonoid standards were analyzed using the same parameters and were used for quantification purposes.

### Bacteria and growth conditions

*F*. *nucleatum* ATCC 25586 was grown under anaerobic conditions (80% N_2_, 10% CO_2_, 10% H_2_) at 37°C in Todd-Hewitt broth (THB; Becton Dickinson and Company, Sparks, MD, USA) supplemented with 0.001% hemin and 0.0001% vitamin K.

### Cell cultures

The previously characterized human oral epithelial cell line GMSM-K [26] was kindly provided by V. Murrah (University of North Carolina, USA). The cells were cultured in Dulbecco’s modified Eagle’s medium (DMEM) supplemented with 10% heat-inactivated fetal bovine serum (FBS) and 100 μg/mL of penicillin G-streptomycin. The previously characterized human oral epithelial cell line B11 [27] was kindly provided by S. Groeger (Justus-Lieig-University Giessen, Germany). The cells were cultured in keratinocyte serum-free medium (K-SFM) supplemented with 50 μg/mL of bovine pituitary extract, 5 ng/mL of recombinant epidermal growth factor, and 100 μg/mL of penicillin G-streptomycin. The human monoblastic leukemia cell line U937 3xκ B-LUC, a subclone of the U937 cell line stably transfected with a luciferase gene coupled to a promoter of three NF-κ B- binding sites [28], was kindly provided by R. Blomhoff (University of Oslo, Norway). These cells were cultured in Roswell Park Memorial Institute 1640 medium (RPMI-1640) supplemented with 10% FBS, 100 μg/mL of penicillin G/streptomycin, and 75 μg/mL of hygromycin B. The cultures were incubated in a humidified incubator with a 5% CO_2_ atmosphere at 37°C. All the culture media and supplements were obtained from Life Technologies Inc. (Burlington, ON, Canada)

### Determination of minimum inhibitory and minimum bactericidal concentrations

The minimum inhibitory concentration (MIC) and minimum bactericidal concentration (MBC) values of the cocoa extract were determined using a broth microdilution assay [29]. A 24-h culture of *F*. *nucleatum* was diluted in fresh medium to an optical density at 660 nm (OD_660_) of 0.1, which corresponds to a concentration of 8 x 10^7^ colony forming units (CFU)/mL. Equal volumes (100 μL) of the bacterial culture and two-fold serial dilutions of the cocoa extract (31.25 to 4,000 μg/mL) in fresh medium were mixed in the wells of a 96-well tissue culture treated, flat-bottom, microplate (Sarstedt, Newton, NC, USA). Wells without bacteria or cocoa extract (but with carrier solvant) were used as controls. The microplate was incubated for 24 h under anaerobic conditions prior to assessing bacterial growth by recording the OD_660_ using a Synergy 2 microplate reader (BioTek Instruments, Winooski, VT, USA). The MIC value was defined as the lowest concentration of the cocoa extract that completely prevented bacterial growth. The MBC value was determined by spreading 5-μL aliquots from each well that exhibited no visible growth on sheep blood-supplemented THB agar plates. The plates were then incubated at 37°C for 48 h in an anaerobic chamber. The MBC value was defined as the lowest concentration at which no colonies formed. The MIC and MBC assays were performed in triplicate in two independent experiments.

### Biofilm formation

The effect of the cocoa extract on biofilm formation by *F*. *nucleatum* was assessed using the 96-well microplate described above after recording bacterial growth. Planktonic and poorly-attached bacterial cells were removed by aspiration with a 26g needle. Biofilms were stained with 100 μL of 0.01% crystal violet for 15 min, washed with distilled water, and dried at 37°C for 2 h. To release biofilm-embedded crystal violet, 100 μL of 75% ethanol was added, and the plate was shaken for 15 min. The biofilm biomass was estimated by recording the absorbance at 550 nm (A_550_) using a Synergy 2 microplate reader. Assays were performed in triplicate in two independent experiments.

### Adherence to oral epithelial cells and to a basement membrane model

The effect of the cocoa extract on the adherence of *F*. *nucleatum* to oral epithelial cells (GMSM-K cell line) and a basement membrane model (Matrigel^TM^; BD Biosciences, Bedford, MA, USA) was investigated, as previously described by Ben Lagha and Grenier [30]. Briefly, bacterial cells from a 24-h culture of *F*. *nucleatum* were labeled with fluorescein isothiocyanate (FITC, 0.03 mg/mL) and were suspended in DMEM medium supplemented with 10% heat-inactivated FBS or 50 mM phosphate-buffered saline (PBS; pH 7) to assess adherence to the epithelial cells or the Matrigel^TM^, respectively. Epithelial cells (1.5 x 10^6^ cells/mL) were seeded at confluence in a 96-well clear bottom black microplate (Greiner Bio-One North America, Monroe, NC, USA), which was incubated (37°C/5% CO_2_) overnight to allow cell adherence. Cell monolayers were pre-incubated (30 min) with two-fold serial dilutions of the cocoa extract (15.63 to 500 μg/mL in DMEM medium) prior to adding FITC-labeled *F*. *nucleatum* cells at a multiplicity of infection (MOI) of 100. The microplate was incubated for a further 4 h at 37°C in a 5% CO_2_ atmosphere. Unbound bacteria were then removed by aspiration, and adhering epithelial cells were washed twice with PBS. The number of bacteria adhered to the epithelial cell monolayers was estimated by determining relative fluorescence units (RFU; excitation wavelength 495 nm, emission wavelength 525 nm) using a Synergy 2 microplate reader. Matrigel^TM^, a basement membrane extract containing several extracellular matrix components, including laminin, type IV collagen, heparin sulfate proteoglycans, and entactin, was diluted 1:10 in ice-cold PBS, was added (100 μL) to the wells of 96-well clear bottom black microplates and was maintained at room temperature for 2 h to allow gellification. The Matrigel^TM^ was then washed twice with PBS, and two-fold serial dilutions of the cocoa extract (15.63 to 500 μg/mL in PBS) were added to the wells. After a 30-min incubation at room temperature, FITC-labeled *F*. *nucleatum* cells (OD_660_ = 0.5) were added to each well (100 μL), and the microplate was incubated at 37°C for 4 h. The number of bacterial cells that adhered to the basement membrane model was determined as described above for the oral epithelial monolayers. Wells without *F*. *nucleatum* were used as controls to measure basal autofluorescence, while wells with no cocoa extract were used to determine 100% adherence values. All the above assays were performed in triplicate in two independent experiments.

### Production of hydrogen sulfide

The production of H_2_S from cysteine and homocysteine by *F*. *nucleatum* was assessed by monitoring the precipitation of bismuth sulfide, according to the procedure described by Yoshida et al. [12], with slight modifications. Cells from a 24-h culture of *F*. *nucleatum* were harvested by centrifugation and were suspended in oxygen-free 0.1% Tryptone to an OD_660_ of 1.0. Aliquots (50 μL) of the bacterial suspension were pipetted into the wells of a 96-well microplate. The cocoa extract (125, 250, 500, 1000 μg/mL) was added (50 μL) to the wells along with 100 μL of a reaction mixture containing 400 mM triethanolamine-HCl, 20 μM pyridoxal-5′-phosphate, 10 mM bismuth III chloride, 10 mM L-cysteine, 10 mM DL-homocysteine, and 20 mM EDTA (pH 8). The plate was sealed with a plastic sheet and was incubated under anaerobic conditions for 1 h at 37°C. H_2_S production was quantified by measuring the absorbance at 620 nm (A_620_) using a BioTek Synergy 2 microplate reader. The control values (without bacteria) were subtracted from the corresponding reactions to compensate for background absorbance by the medium. The assay was performed in triplicate in two independent experiments.

### Determination of cell viability

With the objective to identify non-cytotoxic concentrations of the cocoa extract for the cell lines used below, an MTT (3-[4,5-diethylthiazol-2-yl]-2,5diphenyltetrazolium bromide) colorimetric assay (Roche Diagnostics, Laval, QC, Canada) was performed according to the manufacturer’s protocol. Cell lines were treated (24 h) with two-fold serial dilutions of the cocoa extract (31.25, 62.5, 125, 250, and 500 μg/mL), prior to assess cell viability.

### Cytokine secretion by oral epithelial cells

The human oral epithelial cell line GMSM-K was used to investigate the effect of the cocoa extract on the secretion of the pro-inflammatory cytokines interleukin-6 (IL-6) and IL-8. Epithelial cells were seeded (10^6^ cells/well) in a 6-well microplate and were cultured overnight at 37°C in a 5% CO_2_ atmosphere. The epithelial cells were then pre-treated with non-cytotoxic concentrations of the cocoa extract for 30 min prior to being stimulated with *F*. *nucleatum* at an MOI of 100. After a 24-h incubation, the culture medium supernatants were collected and were stored at -20°C until used. Cells incubated in the absence of the cocoa extract and stimulated or not with *F*. *nucleatum* were used as controls. IL-6 and IL-8 concentrations were determined using ELISA kits (R&D Systems, Minneapolis, MN, USA) according to the manufacturer’s protocol. Assays were performed in triplicate in two independent experiments.

### Activation of the NF-κB transcription factor in monocytes

To determine the effect of the cocoa extract on *F*. *nucleatum*-induced NF-κB activation, the U937 3xκB-LUC monocytes were seeded (10^5^ cells/well) in the wells of a black wall, black bottom, 96-well microplate (Greiner Bio-One North America) and were pre-incubated with non-cytotoxic concentrations of cocoa extract for 30 min. Thereafter, the monocytes were stimulated with *F*. *nucleatum* at an MOI of 100 for 6 h. Wells containing monocytes but no *F*. *nucleatum* or no cocoa extract were used as controls. A commercial inhibitor (BAY-11-7082 [25 μM], EMD Millipore Canada, Mississauga, ON, Canada) of the NF-κB signaling pathway was used as a positive control. Bright-Glo reagent (Promega Corporation, Durham, NC, USA) was used according to the manufacturer’s protocol to measure luciferase activity and determine NF-κB activation. Luminescence was monitored using a Synergy 2 microplate reader. Assays were performed in triplicate in two independent experiments.

### Oral epithelial barrier function

The human oral epithelial cell line B11 was used to investigate the effect of the cocoa extract on epithelial barrier function using the protocol described by Ben Lagha and Grenier [30]. Epithelial cells were seeded on Transwell^TM^ clear polyester membrane inserts (6.5 mm diameter, 0.4 μm pore size; Corning Co., Cambridge, MA, USA) at a concentration of 3 x 10^5^ cells per insert. The basolateral and apical compartments were filled with 0.6 mL and 0.1 mL of culture medium, respectively. After a 72-h incubation (37°C/5% CO_2_), the culture medium was replaced with fresh antibiotic-free K-SFM, and the cells were incubated for a further 16 h. Non-cytotoxic concentrations of the cocoa extract were added to the apical compartment, and the integrity of the epithelial tight junctions was determined by monitoring the transepithelial electrical resistance (TER) using an ohmmeter (EVOM2, World Precision Instruments, Sarasota, FL, USA). TER was recorded after a 0-, 2-, 4-, 8-, 24-, or 48-h incubation (37°C/ 5% CO_2_). Resistance values were calculated in Ohms (Ω)/cm^2^ by multiplying the resistance values by the surface area of the membrane filter. Results were expressed as a percentage of the basal control value measured at time 0 (100% value). Assays were performed in triplicate in two independent experiments.

### Statistical analysis

All experiments were performed in triplicate in two independent experiments, and the means ± standard deviations (SD) were calculated. A one-way ANOVA with a post hoc Bonferroni multiple comparison test was used to analyze the data. Results were considered statistically significant at *p* < 0.01.

## Results

### Polyphenolic composition

The polyphenolic composition of the cocoa extract, as determined by the chromatographic and MS analyses, is reported in Table 1. The fraction contained 30.93% of polyphenols. Phenolic acids, flavonols, anthocyanins, flavan-3-ols, and procyanidins made up 0.15%, 0.72%, 0.12%, 2.19%, and 96.82% of the total polyphenols, respectively. Monomers, dimers, trimers, and tetramers made up 75% of the total procyanidin content.

**Table 1:** Phenolic composition of the cocoa extract.

	Compound	Amount (mg/100 g dry weight)
**PHENOLIC ACIDS**		**47.42**
	Caffeic acid	2.29
	Caffeoyl glucoside	0.42
	Chlorogenic acid	1.42
	*m*-Coumaric acid	0.29
	*p*-Coumaric acid	0.79
	Cryptochlorogenic acid	0.27
	*p*-Hydroxybenzoic acid	0.96
	Protocatechuic acid	35.56
	γ-Resorcyclic acid	4.48
	Shikimic acid	0.94
**FLAVONOLS**		**223.71**
	Kaempferol glucoside / galactoside	0.89
	Phlorizin	2.65
	Quercetin	6.45
	Quercetin 3-arabinoside	0.43
	Quercetin 3-galactoside	85.50
	Quercetin 3-glucoside	34.59
	Quercetin 3-xyloside	91.66
	Rutin	1.54
**ANTHOCYANINS**		**36.66**
	Cyanidin 3-arabinoside / xyloside	21.92
	Cyanidin 3-galactoside / glucoside	14.35
	Cyanidin 3-sambubioside	0.39
**FLAVAN-3-OLS**		**676.36**
	Catechin	150.23
	Epicatechin	526.13
**PROCYANIDINS**		**29943**
	Monomers	6119
	Dimers	7838
	Trimers	4823
	Tetramers	3794
	Pentamers	2027
	Hexamers	1759
	Heptamers	887
	Octamers	647
	Nonamers	656
	Decamers	1074
	Polymers (DP > 10)	319

DP: Degree of polymerization.

### Effects on *F*. *nucleatum* growth and virulence properties

**Fig 1:**
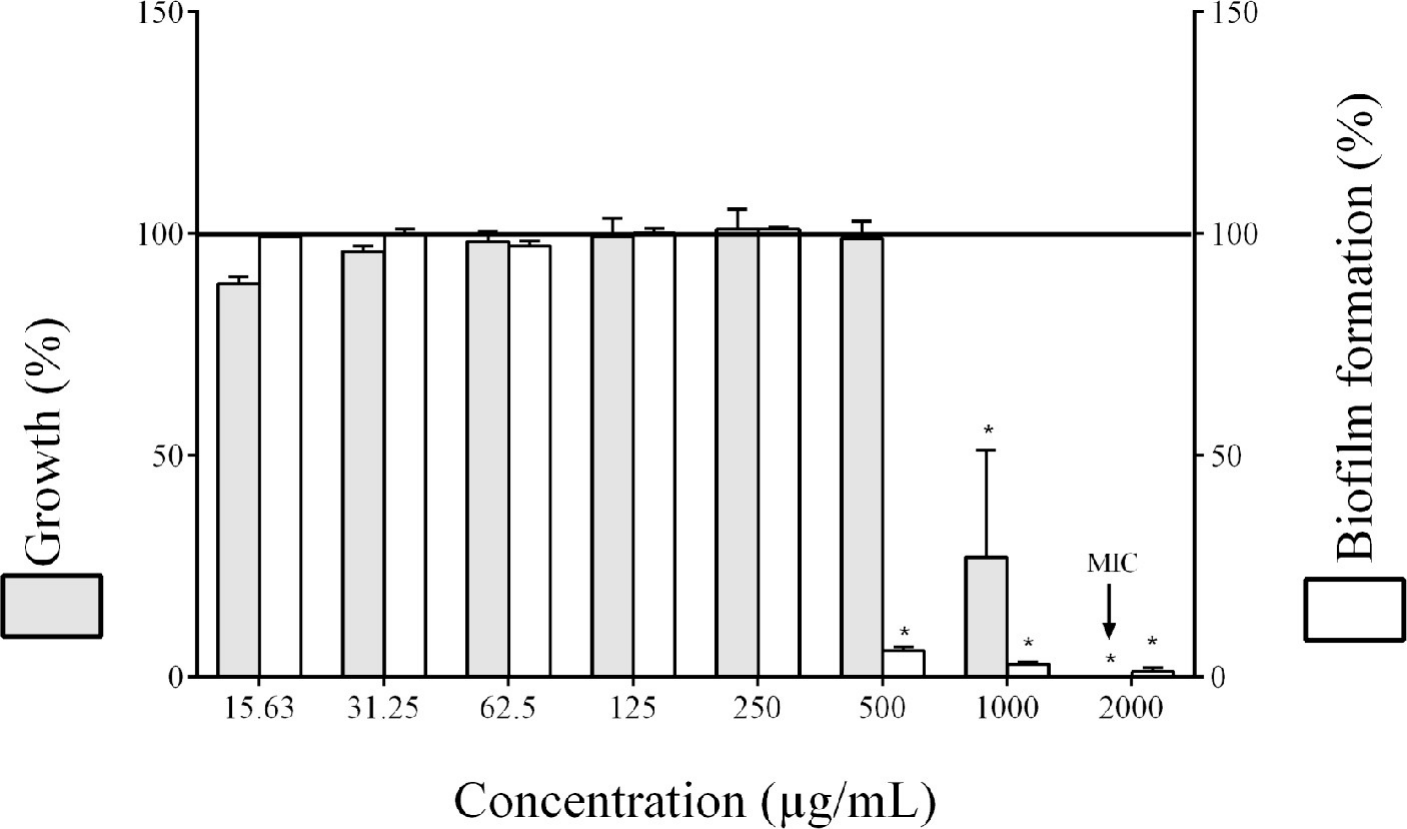
Effect of the cocoa extract on *F*. *nucleatum* growth and biofilm formation. A relative value of 100% was assigned to growth and biofilm formation in the absence of the cocoa extract. MIC: minimum inhibitory concentration. *, significant inhibition (*p* < 0.01) compared to the control (no cocoa extract).

The antibacterial activity of the cocoa extract was assessed using a broth microdilution assay. As shown in Fig 1, the cocoa extract had a MIC value of 2000 μg/mL against *F*. *nucleatum* while a concentration of 1000 μg/mL reduced bacterial growth by 73.6%. No MBC value was obtained, suggesting that the cocoa extract has a bacteriostatic mode of action, although concentrations > 2000 μg/mL may have a bactericidal effect. Interestingly, the cocoa extract prevented biofilm formation by *F*. *nucleatum*. When cultivated in the presence of 500 μg/mL of the cocoa extract, the growth of *F*. *nucleatum* was not affected while the biofilm was reduced by 94.3% (Fig 1).

**Fig 2:**
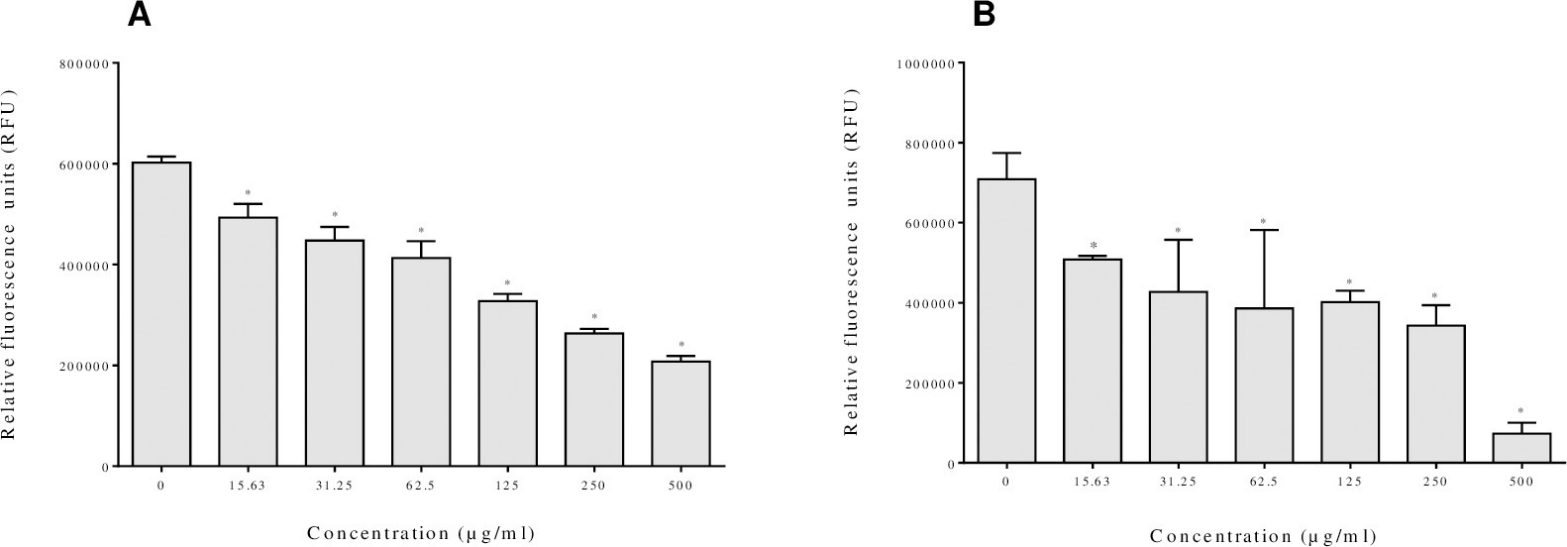
Effect of the cocoa extract on the adherence of *F*. *nucleatum* to oral epithelial cells (GMSM-K cell line) (Panel A) and the Matrigel™ basement membrane model (Panel B). *, significant inhibition (*p* < 0.01) compared to the control (no cocoa extract).

The ability of the cocoa extract to reduce the adherence of *F*. *nucleatum* to oral epithelial cells was investigated. As shown in Fig 2A, the cocoa extract dose-dependently inhibited bacterial adherence to oral epithelial cells. At the lowest (15.63 μg/mL) and the highest (500 μg/mL) concentrations of the cocoa extract tested, the adherence of *F*. *nucleatum* was reduced by 17.8% and 65.7%, respectively. We then evaluated the capacity of the cocoa extract to attenuate the adherence of *F*. *nucleatum* to the Matrigel^TM^ basement membrane model. The cocoa extract also exerted a dose-dependent inhibitory effect on adherence to oral epithelial cells. At 15.63 μg/mL, the cocoa extract reduced the adherence of *F*. *nucleatum* to the Matrigel^TM^ basement membrane model by 27.9%, while a concentration of 500 μg/mL inhibited adherence by 89.7% (Fig 2B).

The effect of the cocoa extract on H_2_S production by *F*. *nucleatum* was determined with a colorimetric assay using cysteine and homocysteine as substrates. As reported in Table 2, the extract dose-dependently reduced H_2_S production. At the highest concentration tested (1000 μg/mL), the cocoa extract reduced the production of H_2_S by 71.4%.

**Table 2:** Effect of the cocoa extract on H_2_S production by *F*. *nucleatum*.

Cocoa extract (μg/mL)	H_2_S production (%)*
0	100 ± 9.7
125	64.3 ± 7.7 †
250	51.7 ± 14.3 †
500	35.5 ± 9.8 †
1000	28.6 ± 11.4 †

A value of 100% was assigned to H_2_S production by *F*. *nucleatum* in the absence of the extract.

*: Results are expressed as the means ± SD of triplicate assays from two independent experiments. A value of 100% was assigned to the control (no cocoa extract).

†, Significantly different (*p* < 0.01) from the control (no cocoa extract).

In summary, the cocoa extract, in addition to inhibit the growth of *F*. *nucleatum*, can impair its virulence determinants, including biofilm formation, adherence properties and H_2_S production.

### Effects on *F*. *nucleatum*-induced inflammatory response

**Fig 3:**
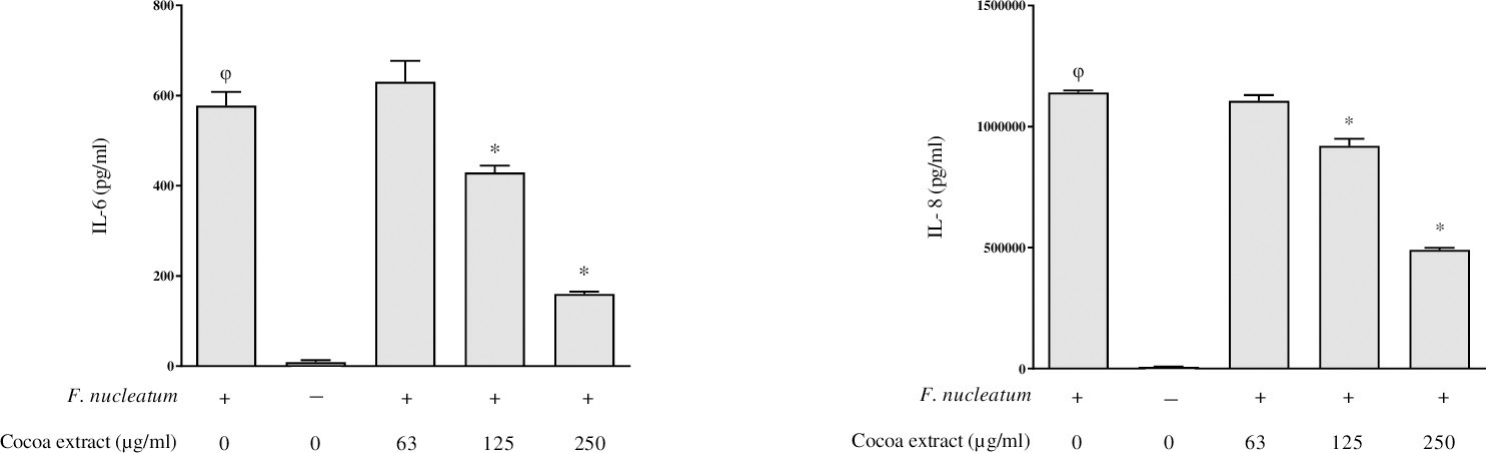
Effect of the cocoa extract on the secretion of IL-6 and IL-8 by oral epithelial cells (GMSM-K cell line) stimulated with *F*. *nucleatum* (MOI = 100). φ, significant increase compared to the control (no *F*. *nucleatum* stimulation). *, significant inhibition (*p* < 0.01) compared to the control (no cocoa extract).

In order to explore the anti-inflammatory property of the cocoa extract, we investigated its ability to attenuate the secretion of IL-6 and IL-8 by oral epithelial cells (GMSM-K cell line) challenged with *F*. *nucleatum*. At concentrations up to 250 μg/mL, the cocoa extract did not significantly reduce cell viability (Table 3). As shown in Fig 3, *F*. *nucleatum* induced a significant increase in the secretion of both cytokines by oral epithelial cells. This increased secretion was dose-dependently reduced in the presence of the cocoa extract. More specifically, 250 μg/mL of the cocoa extract reduced the secretion of IL-6 and IL-8 by 71.9% and 57.6%, respectively.

**Table 3:** Effects of the cocoa extract on viability of B11 oral epithelial cells, GMSM-K oral epithelial cells, and U937 3xκB-LUC monocytic cells.

Cocoa extract (μg/mL)	% Viability (Mean ± standard deviation)		
	B11 cell line	GMSM-K cell line	3xκB-LUC cell line
None	100 ± 3.5	100 ± 4.4	100 ± 6.6
500	61.4 ± 0.2*	46.1 ± 15.6*	39.7 ± 8.7*
250	79.5 ± 11.1*	89.8 ± 12.5	53.4 ± 9.6*
125	121.2 ± 8.8	109.7 ± 17.7	94.5 ± 8.3
62.5	151.3 ± 9.5	130.2 ± 5.5	111.7 ± 6.5
31.25	153.8 ± 8.5	122.2 ± 16.8	113.4 ± 10.2

Cell viability following a 24-h treatment was assessed using an MTT (3-[4,5-diethylthiazol-2-yl]-2,5diphenyltetrazolium bromide) colorimetric assay.

*, Significant decrease (*p* < 0.01) in cell viability compared to untreated control cells.

**Fig 4:**
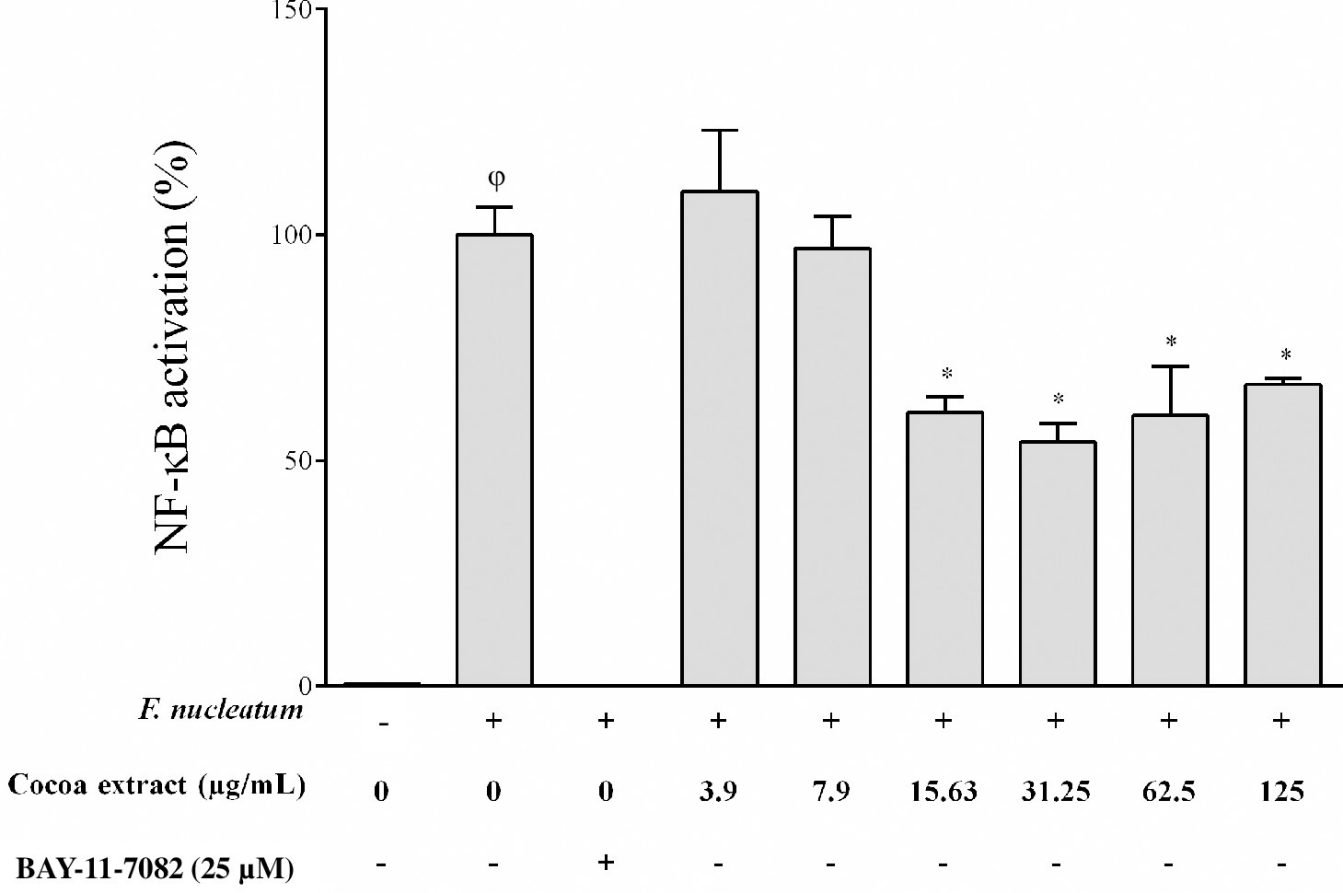
Effect of the cocoa extract on the activation of the NF-κB signaling pathway induced by *F*. *nucleatum* (MOI = 100). A value of 100% was assigned to the activation obtained with *F*. *nucleatum* in the absence of the cocoa extract. The commercial inhibitor BAY-11-7082 was used as a positive inhibitory control. φ, significant increase compared to the control (no *F*. *nucleatum* stimulation). *, significant inhibition (*p* < 0.01) compared to the control (no cocoa extract).

We then used a second model to confirm the anti-inflammatory property of the cocoa extract. Given that the NF-κB signalling pathway is a transcription factor involved in inflammatory processes leading to the production of cytokines and chemokines, we evaluated the effect of the cocoa extract on NF-κB activation using the U937-3xκB monocytic cell line. The cocoa extract at concentrations up to 125 μg/mL did not significantly reduce cell viability (Table 3). As shown in Fig 4, the cocoa extract reduced *F*. *nucleatum*-induced NF-κB activation, although less efficiently than the commercial positive inhibitory control (BAY-11-7082). Significant inhibitory effects (33.1% to 45.8%) were observed at concentrations ranging from 15.63 to 125 μg/mL.

In summary, the cocoa extract exerts anti-inflammatory properties towards two cell types challenged with *F*. *nucleatum*. It attenuates cytokine secretion by oral epithelial cells and decreases the activation of the NF-κB signalling pathway in monocytes.

### Effects on oral epithelial barrier function

**Fig 5:**
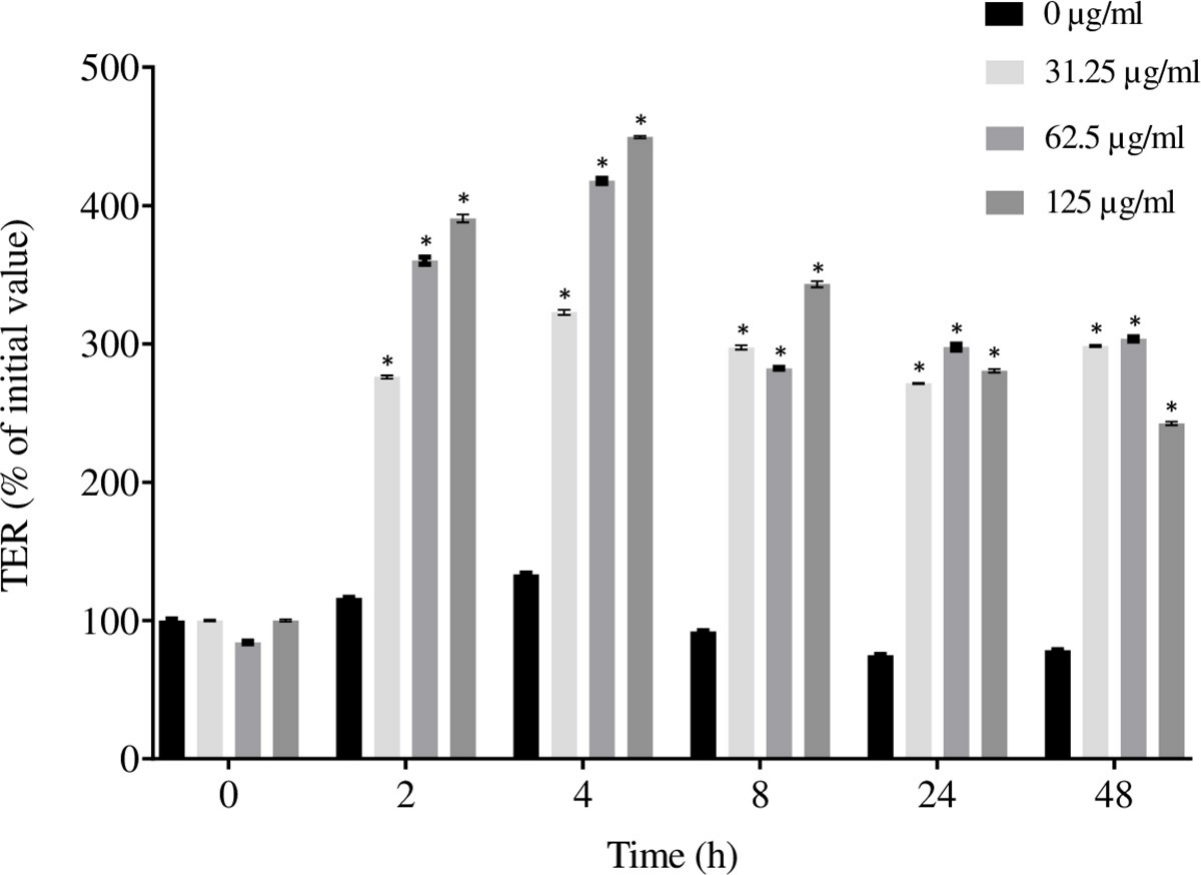
Effect of the cocoa extract on oral epithelial tight junction integrity. Time- and dose-dependent effects of the cocoa extract on TER values in oral epithelial cells (B11 cell line). A 100% value was assigned to the TER value at time 0. *, significant increase (*p* < 0.01) compared to the control (no cocoa extract).

As the tight junctions of oral epithelial cells likely play a key role in protecting the underlying connective tissues from invasion by periodontopathogens, we investigated the ability of the cocoa extract to enhance oral epithelial barrier function. We monitored, over a 48-h period, the TER values of a monolayer of epithelial cells (B11 cell line) exposed to the cocoa extract at concentrations (≤ 125 μg/mL) that did not affect cell viability (Table 3). As shown in Fig 5, the cocoa extract caused a significant time- and dose-dependent increase in TER values. The TER values reached their maximum after a 4-h treatment of the cells and tended to decrease thereafter. Despite this decrease, the TER values of the treated cells remained significantly higher than the untreated control cells. Following a 4-h exposure to 31.25, 62.5, and 125 μg/mL of the cocoa extract, the TER values increased 2.5-, 3.2-, and 3.5-fold, respectively, compared to the untreated control cells.

## Discussion

Periodontal disease is initiated by a synergistic and dysbiotic polymicrobial community colonizing the subgingival areas [31]. *F*. *nucleatum* is a key pathobiont involved in periodontal biofilm formation by bridging the early and late colonizers [4]. It expresses several virulence factors that allow it to adhere to and invade oral epithelial cells and trigger an immune response [5, 6]. The focus of the present study was to evaluate the antimicrobial and anti-inflammatory activities of a cocoa extract against *F*. *nucleatum*. Given the important role of the oral epithelium as a physical barrier to prevent bacterial invasion of gingival connective tissues [17], we also investigated the ability of the cocoa extract to enhance epithelial barrier function. To the best of our knowledge, these aspects have never previously been studied.

Cocoa phytochemical constituents, particularly polyphenols, exhibit beneficial health properties and thus have the capacity to improve and prevent many human diseases [22–24]. However, the potential oral health-promoting benefits of cocoa have been poorly studied to date. In terms of the antibacterial effects of cocoa polyphenols against cariogenic bacteria, Percival et al. [32] reported that, while the growth of *Streptococcus sanguinis* was inhibited, a cocoa bean extract did not affect the growth of *Streptococcus mutans*. Cocoa polyphenols have also been reported to inhibit acid production and biofilm formation by and the glycosyltransferase activity of *S*. *mutans* [32, 33]. Hirao et al. [34] showed that a crude cocoa polyphenol extract can reduce the viability of *F*. *nucleatum*, *P*. *gingivalis*, and *Prevotella intermedia*. In the present study, we showed that a cocoa extract completely prevents the growth of *F*. *nucleatum* when added to the culture medium at a concentration of 2000 μg/mL. The exact antibacterial mechanism of action of the cocoa extract on *F*. *nucleatum* is difficult to elucidate given that the fraction contains a variety of polyphenols, which may act on different targets. However, growth inhibition may be related to the ability of epicatechin, which is the major phenolic compound in the cocoa extract, to strongly bind to the lipid bilayer of the bacterial membrane, resulting in a loss of cell structure and function, leading to cell death [35].

The adherence of bacteria to oral surfaces is the first step in biofilm formation and host colonization. In the present study, we showed that *F*. *nucleatum* adheres to oral epithelial cells as well as to the Matrigel™ basement membrane model. The cocoa extract significantly inhibited the ability of *F*. *nucleatum* to adhere to both surfaces. The results of a colorimetric microplate assay showed that the cocoa extract almost completely prevented biofilm formation by *F*. *nucleatum* at a concentration (500 μg/mL) that did not reduce bacterial growth. As *F*. *nucleatum* is a critical bacterium involved in subgingival biofilm formation, the above observation suggests that the cocoa extract may prevent or slow periodontal disease initiation and progression since biofilms enable bacteria to evade immune defenses and resist mechanical removal and chemotherapeutic agents.

*F*. *nucleatum* is known to produce large quantities of H_2_S and consequently has been associated with halitosis [12]. Moreover, H_2_S is a metabolite that can induce an inflammatory response and that has a toxic effect on oral epithelial cells and gingival fibroblasts [36–39]. The cocoa extract significantly reduced H_2_S production by *F*. *nucleatum*, suggesting that it may be of interest for reducing unpleasant oral odors as well as gingival inflammation.

*F*. *nucleatum* induced the production of IL-8 and IL-6 by the GMSM-K cell line, which is in agreement with previous studies [40, 41]. An imbalance in IL-8 levels can contribute to the transmigration of neutrophils from the submucosa to the periodontal pocket and can, consequently, lead to exaggerated inflammation and periodontal tissue destruction [42, 43]. On the other hand, prolonged and elevated secretion of IL-6 may induce periodontal bone loss in patients with chronic periodontitis [44] by osteoclast activation caused by the overexpression of RANKL [45, 46]. Given this, the modulation of cytokine expression in gingival tissues during periodontal disease may be key for maintaining periodontal health. Interestingly, the cocoa extract dose-dependently reduced the secretion of both IL-6 and IL-8. Consistent with these results, previous studies have shown that cocoa causes a reduction in IL-8 levels in inflammatory models of colonic epithelial cells [47, 48].

The NF-κB pathway is a key transcription factor that plays a crucial role in the innate immune response and in chronic inflammation [49, 50]. It can induce the expression of inflammatory mediators, and its dysregulation contributes to the chronic inflammatory process [51, 52]. We used the U937-3xκ B-LUC cell line to show that the cocoa extract significantly inhibits the activation of NF-κB. In agreement with our results, polyphenols identified in cocoa-related products exhibit beneficial effects with respect to cardiovascular inflammatory disorders and changes in the levels of pro-inflammatory cytokines associated with preeclampsia by inhibiting NF-κB activation [53, 54]. In this respect, the (−)-epicatechin, (+)-catechin, and dimeric procyanidins B1 and B2 in *T*. *cacao* seem to play an anti-inflammatory role due to their capacity to modulate NF-κB binding to DNA [55–57].

The ability of periodontal pathogens to damage the epithelial barrier and translocate into gingival connective tissues contributes to the destruction of the periodontal support [58, 59]. In the present study, we used an *in vitro* epithelial barrier model to investigate the capacity of the cocoa extract to enhance epithelial barrier function. Our results showed that the cocoa extract has a positive effect on tight junction integrity by increasing the TER values. This is in agreement with Bitzer et al. [47], who reported that polymeric cocoa procyanidins can prevent epithelial inflammation and the loss of gut barrier function in an *in vitro* model of colonic inflammation.

**Fig 6:**
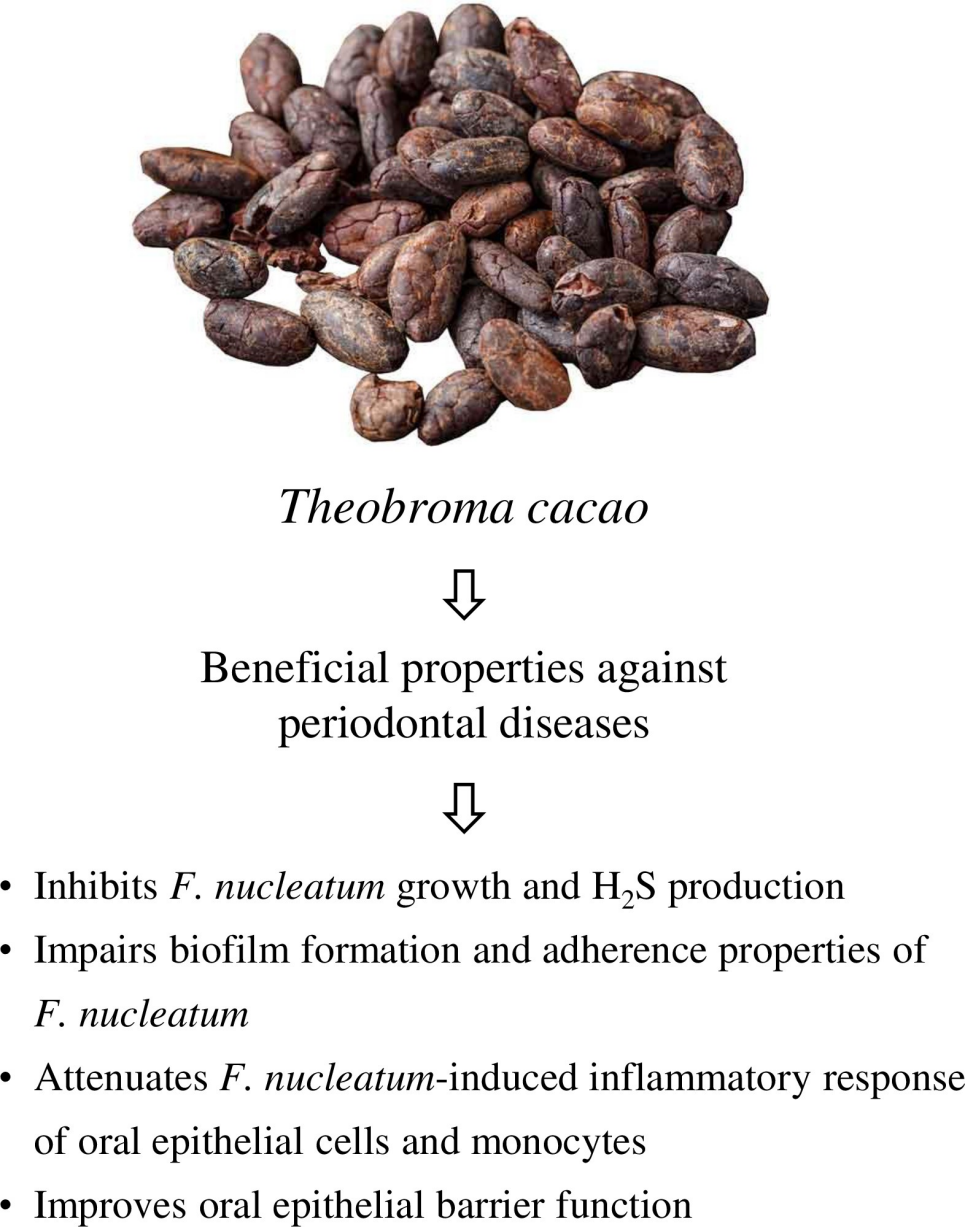
Beneficial effects of the cocoa extract against periodontal diseases.

In summary, this study brought evidence that the cocoa extract under investigation acts on multiple targets related to the etiology of periodontal diseases (Fig 6). On the one hand, while at a high concentration it inhibits the growth of *F*. *nucleatum*, lower concentrations that do not reduce growth, it reduces biofilm formation and adherence properties. This is of interest given that the objective of anti-adherence therapies is to inhibit the host colonization without affecting bacterial viability, thus minimizing the emergence of resistant strains. On the other hand, the cocoa extract decreases the inflammatory response of mucosal and immune cell types and can thus attenuate periodontal tissue destruction.

Within the limitations of the present study, cocoa polyphenols, incorporated into mouthrinses, toothpastes, gels, or local drug delivery systems, can be considered as a promising therapeutic agent for the prevention and/or treatment of periodontal disease due to its antimicrobial and anti-inflammatory properties as well as its ability to enhance the epithelial barrier protective function. Interestingly, Tomofuji et al. [60] used a rat-periodontitis model and reported that a cocoa-enriched diet could inhibit alveolar bone loss and polymorphonuclear leukocyte infiltration. Further research to evaluate the clinical efficacy of cocoa polyphenols is needed.

## References

[1] Papapanou PN, Sanz M, Buduneli N, Dietrich T, Feres M, Fine DH, et al. Periodontitis: Consensus report of workgroup 2 of the 2017 world workshop on the classification of periodontal and peri-implant diseases and conditions. J Periodontol. 2018;89: S173–S182. 10.1002/JPER.17-0721 29926951

[2] Curtis MA, Diaz PI, Van Dyke TE. The role of the microbiota in periodontal disease. Periodontol 2000. 2020;83: 14–25. 10.1111/prd.12296 32385883

[3] Dahlen G, Basic A, Bylund J. Importance of virulence factors for the persistence of oral bacteria in the inflamed gingival crevice and in the pathogenesis of periodontal disease. J Clin Med. 2019;8: 1339. 10.3390/jcm8091339 31470579 PMC6780532

[4] Kolenbrander PE. Oral microbial communities: biofilms, interactions, and genetic systems. Annu Rev Microbiol. 2000;54: 413–437. 10.1146/annurev.micro.54.1.413 11018133

[5] Han YW. *Fusobacterium nucleatum*: a commensal-turned pathogen. Curr Opin Microbiol. 2015;23: 141–147. 10.1016/j.mib.2014.11.013 25576662 PMC4323942

[6] Brennan CA, Garrett WS. *Fusobacterium nucleatum*—symbiont, opportunist and oncobacterium. Nat Rev Microbiol. 2019;17: 156–166. 10.1038/s41579-018-0129-6 30546113 PMC6589823

[7] Ximénez‐Fyvie LA, Haffajee AD, Socransky SS. Comparison of the microbiota of supra‐ and subgingival plaque in health and periodontitis. J Clin Periodontol. 2000;27: 648–657. 10.1034/j.1600-051x.2000.027009648.x 10983598

[8] Kaplan CW, Lux R, Haake SK, Shi W. The *Fusobacterium nucleatum* outer membrane protein RadD is an arginine-inhibitable adhesin required for inter-species adherence and the structured architecture of multispecies biofilm. Mol Microbiol. 2009;71: 35–47. 10.1111/j.1365-2958.2008.06503.x 19007407 PMC2741168

[9] Han YW, Ikegami A, Rajanna C, Kawsar HI, Zhou Y, Li M, et al. Identification and characterization of a novel adhesin unique to oral fusobacteria. J Bacteriol. 2005;187: 5330–5340. 10.1128/JB.187.15.5330-5340.2005 16030227 PMC1196005

[10] Coppenhagen-Glazer S, Sol A, Abed J, Naor R, Zhang X, Han YW, et al. Fap2 of *Fusobacterium nucleatum* is a galactose-inhibitable adhesin involved in coaggregation, cell adhesion, and preterm birth. Infect Immun. 2015;83: 1104–1113. 10.1128/IAI.02838-14 25561710 PMC4333458

[11] Gaetti-Jardim Júnior E, Avila-Campos MJ. Haemagglutination and haemolysis by oral *Fusobacterium nucleatum*. New Microbiol. 1999;22: 63–67. 10190119

[12] Yoshida A, Yoshimura M, Ohara N, Yoshimura S, Nagashima S, Takehara T, et al. Hydrogen sulfide production from cysteine and homocysteine by periodontal and oral bacteria. J Periodontol. 2009;80: 1845–1851. 10.1902/jop.2009.090012 19905954

[13] Bodet C, Chandad F, Grenier D. Anti-inflammatory activity of a high-molecular-weight cranberry fraction on macrophages stimulated by lipopolysaccharides from periodontopathogens. J Dent Res. 2006;85: 235–239. 10.1177/154405910608500306 16498070

[14] Stathopoulou PG, Benakanakere MR, Galicia JC, Kinane DF. Epithelial cell pro‐inflammatory cytokine response differs across dental plaque bacterial species. J Clin Periodontol. 2010;37: 24–29. 10.1111/j.1600-051X.2009.01505.x 20096064 PMC2900159

[15] Kang MS, Jang HS, Oh JS, Yang KH, Choi NK, Lim HS, et al. Effects of methyl gallate and gallic acid on the production of inflammatory mediators interleukin‐6 and interleukin‐8 by oral epithelial cells stimulated with *Fusobacterium nucleatum*. J Microbiol. 2009;47: 760–767. 10.1007/s12275-009-0097-7 20127471

[16] Mahtout H, Chandad F, Rojo JM, Grenier D. 2011. *Fusobacterium nucleatum* binding to complement regulatory protein CD46 modulates the expression and secretion of cytokines and matrix metalloproteinases by oral epithelial cells. J Periodontol. 2011;82: 311–319. 10.1902/jop.2010.100458 20843232

[17] Groeger SE, Meyle J. Epithelial barrier and oral bacterial infection. Periodontol 2000. 2015;69: 46–67. 10.1111/prd.12094 26252401

[18] Dixon DR, Bainbridge BW, Darveau RP. Modulation of the innate immune response within the periodontium. Periodontol 2000. 2004;35: 53–74. 10.1111/j.0906-6713.2004.003556.x 15107058

[19] He Y, Shiotsu N, Uchida-Fukuhara Y, Guo J, Weng Y, Ikegame M, et al. Outer membrane vesicles derived from *Porphyromonas gingivalis* induced cell death with disruption of tight junctions in human lung epithelial cells. Arch Oral Biol. 2020;118: 104841. 10.1016/j.archoralbio.2020.104841 32717445

[20] Ji S, Choi YS, Choi Y. Bacterial invasion and persistence: critical events in the pathogenesis of periodontitis? J Periodontal Res. 2015;50: 570–585. 10.1111/jre.12248 25487426

[21] Graziani F, Karapetsa D, Alonso B, Herrera D. Nonsurgical and surgical treatment of periodontitis: how many options for one disease? Periodontol 2000. 2017;75: 152–188. 10.1111/prd.12201 28758300

[22] Andújar I, Recio MC, Giner RM, Ríos JL. Cocoa polyphenols and their potential benefits for human health. Oxid Med Cell Longev. 2012;2012: 906252. 10.1155/2012/906252 23150750 PMC3488419

[23] Lamuela-Raventos RM, Romero-Pérez AI, Andrés-La Cueva C, Tornero A. Health effects of cocoa flavonoids. Food Sci Tech Int. 2005;11: 159–176.

[24] De Araujo QR, Gattward JN, Almoosawi S, Silva Md, Dantas PA, De Araujo Júnior QR. Cocoa and human health: From head to foot- a review. Crit Rev Food Sci Nutr. 2016;56: 1–12. 10.1080/10408398.2012.657921 24915376

[25] Dudonne S, Dube P, Pilon G, Marette A, Jacques H, Weisnagel J, et al. Modulation of strawberry/cranberry phenolic compounds glucuronidation by co-supplementation with onion: Characterization of phenolic metabolites in rat plasma using an optimized muSPE-UHPLC-MS/MS method. J Agric Food Chem. 2014;62: 3244–3256. 10.1021/jf404965z 24628392

[26] Gilchrist EP, Moyer MP, Shillitoe EJ, Clare N, Murrah VA. Establishment of a human polyclonal oral epithelial cell line. Oral Surg Oral Med Oral Pathol Oral Radiol Endod. 2000;90: 340–347. 10.1067/moe.2000.107360 10982956

[27] Gröeger S, Michel J, Meyle J. Establishment and characterization of immortalized human gingival keratinocyte cell lines. J Periodontal Res. 2008;43: 604–614. 10.1111/j.1600-0765.2007.01019.x 18771458

[28] Carlsen H, Moskaug JØ, Fromm SH, Blomhoff R. *In vivo* imaging of NF-kappa B activity. J Immunol. 2002;168: 1441–1446. 10.4049/jimmunol.168.3.1441 11801687

[29] Ben Lagha A, LeBel G, Grenier D. Tart cherry (*Prunus cerasus* L.) fractions inhibit biofilm formation and adherence properties of oral pathogens and enhance oral epithelial barrier function. Phytother Res. 2020;34: 886–895. 10.1002/ptr.6574 31846135

[30] Ben Lagha A, Grenier D. Black tea theaflavins attenuate *Porphyromonas gingivalis* virulence properties, modulate gingival keratinocyte tight junction integrity and exert anti-inflammatory activity. J Periodontal Res. 2017;52: 458–470. 10.1111/jre.12411 27549582

[31] Hajishengallis G, Lamont RJ. Beyond the red complex and into more complexity: the polymicrobial synergy and dysbiosis (PSD) model of periodontal disease etiology. Mol Oral Microbiol. 2012;27: 409–419. 10.1111/j.2041-1014.2012.00663.x 23134607 PMC3653317

[32] Percival RS, Devine DA, Duggal MS, Chartron S, Marsh PD. The effect of cocoa polyphenols on the growth, metabolism, and biofilm formation by *Streptococcus mutans* and *Streptococcus sanguinis*. Eur J Oral Sci. 2006;114: 343–348. 10.1111/j.1600-0722.2006.00386.x 16911106

[33] Osawa K, Miyazaki K, Shimura S, Okuda J, Matsumoto M, Ooshima T. Identification of cariostatic substances in the cacao bean husk: their anti-glucosyltransferase and antibacterial activities. J Dent Res. 2001;80: 2000–2004. 10.1177/00220345010800111001 11759010

[34] Hirao C, Nishimura e, Kamei M, Ohshima T, Maeda N. Antibacterial effects of cocoa on periodontal pathogenic bacteria. J Oral Biosci. 2010; 52: 283–291.

[35] Koech KR, Wachira FN, Ngure RN, Wanyoko JK, Bii CC, Karori SM, et al. 2013. Antimicrobial, synergistic and antioxidant activities of tea polyphenols. In: Mendez-Vilas A, editor. Microbial pathogens and strategies for combating them: science, technology and education. Badajoz: Formatex Research Center. P. 971–981.

[36] Chen W, Kajiya M, Giro G, Ouhara K, Mackler HE, Mawardi H, et al. Bacteria-derived hydrogen sulfide promotes IL-8 production from epithelial cells. Biochem Biophys Res Commun. 2010;391: 645–650. 10.1016/j.bbrc.2009.11.113 19932683 PMC2874960

[37] Murata T, Yaegaki K, Qian W, Herai M, Calenic B, Imai T, et al. Hydrogen sulfide induces apoptosis in epithelial cells derived from human gingiva. J Breath Res. 2008;2: 017007. 10.1088/1752-7155/2/1/017007 21386151

[38] Zhang JH, Dong Z, Chu L. Hydrogen sulfide induces apoptosis in human periodontium cells. J Periodontal Res. 2010;45: 71–78. 10.1111/j.1600-0765.2009.01202.x 19602114

[39] Yaegaki K, Qian W, Murata T, Imai T, Sato T, Tanaka T, et al. Oral malodorous compound causes apoptosis and genomic DNA damage in human gingival fibroblasts. J Periodontal Res. 2008;43: 391–399. 10.1111/j.1600-0765.2007.01052.x 18942188

[40] Tonetti MS, Imboden MA, Gerber L, Lang NP, Laissue J, Mueller C. Localized expression of mRNA for phagocyte-specific chemotactic cytokines in human periodontal infections. Infect Immun. 1994;62: 4005–4014. 10.1128/IAI.62.9.4005-4014.1994 8063420 PMC303060

[41] Stathopoulou PG, Benakanakere MR, Galicia JC, Kinane DF. Epithelial cell pro-inflammatory cytokine response differs across dental plaque bacterial species. J Clin Periodontol. 2010;37: 24–29. 10.1111/j.1600-051X.2009.01505.x 20096064 PMC2900159

[42] Tonetti MS, Imboden MA, Lang NP. Neutrophil migration into the gingival sulcus is associated with transepithelial gradients of interleukin-8 and ICAM-1. J Periodontol. 1998;69: 1139–1147. 10.1902/jop.1998.69.10.1139 9802714

[43] Darveau RP. The oral microbial consortium’s interaction with the periodontal innate defense system. DNA Cell Biol. 2009;28: 389–395. 10.1089/dna.2009.0864 19435427 PMC2883565

[44] Takahashi K, Takashiba S, Nagai A, Takigawa M, Myoukai F, Kurihara H, et al. Assessment of interleukin-6 in the pathogenesis of periodontal disease. J Periodontol. 1994;65: 147–153. 10.1902/jop.1994.65.2.147 8158511

[45] Mori T, Miyamoto T, Yoshida H, Asakawa M, Kawasumi M, Kobayashi T, et al. IL-1β and TNFα-initiated IL-6-STAT3 pathway is critical in mediating inflammatory cytokines and RANKL expression in inflammatory arthritis. Int Immunol. 2011;23: 701–712. 10.1093/intimm/dxr077 21937456

[46] Mihara M, Hashizume M, Yoshida H, Suzuki M, Shiina M. IL-6/IL-6 receptor system and its role in physiological and pathological conditions. Clin Sci (Lond). 2012;122: 143–159. 10.1042/CS20110340 22029668

[47] Bitzer ZT, Glisan SL, Dorenkott MR, Goodrich KM, Ye L, O’Keefe SF, et al. Cocoa procyanidins with different degrees of polymerization possess distinct activities in models of colonic inflammation. J Nutr Biochem. 2015;26: 827–831. 10.1016/j.jnutbio.2015.02.007 25869594 PMC4469546

[48] Rossin D, Barbosa-Pereira L, Iaia N, Sottero B, Danzero AC, Poli G, et al. Protective effect of cocoa bean shell against intestinal damage: an example of byproduct valorization. Antioxidants (Basel). 2021;10: 280. 10.3390/antiox10020280 33673085 PMC7918452

[49] Hoffmann JA, Kafatos FC, Janeway CA, Ezekowitz RA. Phylogenetic perspectives in innate immunity. Science. 1999;284: 1313–1318. 10.1126/science.284.5418.1313 10334979

[50] Ghosh S, May MJ, Kopp EB. NF-kappa B and Rel proteins: evolutionarily conserved mediators of immune responses. Annu Rev Immunol. 1998;16: 225–260. 10.1146/annurev.immunol.16.1.225 9597130

[51] Gupta SC, Sundaram C, Reuter S, Aggarwal BB. Inhibiting NF-κB activation by small molecules as a therapeutic strategy. Biochim Biophys Acta. 2010;1799: 775–787. 10.1016/j.bbagrm.2010.05.004 20493977 PMC2955987

[52] Liu T, Zhang L, Joo D, Sun SC. NF-κB signaling in inflammation. Signal Transduct Target Ther. 2017;2: 17023. 10.1038/sigtrans.2017.23 29158945 PMC5661633

[53] Zeng H, Locatelli M, Bardelli C, Amoruso A, Coisson JD, Travaglia F, et al. Anti-inflammatory properties of clovamide and *Theobroma cacao* phenolic extracts in human monocytes: evaluation of respiratory burst, cytokine release, NF-κB activation, and PPARγ modulation. J Agric Food Chem. 2011;59: 5342–5350. 10.1021/jf2005386 21486087

[54] Rahayu B, Baktiyani SC, Nurdiana N. *Theobroma* cacao increases cells viability and reduces IL-6 and sVCAM-1 level in endothelial cells induced by plasma from preeclamptic patients. Pregnancy Hypertens. 2016;6: 42–46. 10.1016/j.preghy.2016.01.001 26955771

[55] Selmi C, Mao TK, Keen CL, Schmitz HH, Eric Gershwin M. The anti-inflammatory properties of cocoa flavanols. J Cardiovasc Pharmacol. 2006;47: S163–176. 10.1097/00005344-200606001-00010 16794453

[56] Mackenzie GG, Delfino JM, Keen CL, Fraga CG, Oteiza PI. Dimeric procyanidins are inhibitors of NF-kappaB-DNA binding. Biochem Pharmacol. 2009;78: 1252–1262. 10.1016/j.bcp.2009.06.111 19591805

[57] Mao TK, van de Water J, Keen CL, Schmitz HH, Gershwin ME. Modulation of TNF-alpha secretion in peripheral blood mononuclear cells by cocoa flavanols and procyanidins. Dev Immunol. 2002;9: 135–141. 10.1080/1044667031000137601 12885154 PMC2276101

[58] Andrian E, Grenier D, Rouabhia M. *In vitro* models of tissue penetration and destruction by *Porphyromonas gingivalis*. Infect Immun. 2004;72: 4689–4698. 10.1128/IAI.72.8.4689-4698.2004 15271930 PMC470627

[59] Groeger S, Doman E, Chakraborty T, Meyle J. Effects of *Porphyromonas gingivalis* infection on human gingival epithelial barrier function in vitro. Eur J Oral Sci. 2010;118: 582–589. 10.1111/j.1600-0722.2010.00782.x 21083619

[60] Tomofuji T, Ekuni D, Irie K, Azuma T, Endo Y, Tamaki N, et al. Preventive effects of a cocoa-enriched diet on gingival oxidative stress in experimental periodontitis. J Periodontol. 2009;80: 1799–1808. 10.1902/jop.2009.090270 19905949

